# Influence of maturity, smoking, and drying of fresh maize on sensory acceptability and nutritional content of the developed porridges

**DOI:** 10.1002/fsn3.838

**Published:** 2018-10-10

**Authors:** Lizzie Saka, William Kasapila, Tinna A. Ng'ong'ola Manani, Vincent Mlotha

**Affiliations:** ^1^ Department of Food Science and Technology Lilongwe University of Agriculture and Natural Resources Lilongwe Malawi

**Keywords:** acceptability, Fresh maize processing, maize–soy flour blends, porridge, smoke drying

## Abstract

This study investigated the potential of using the underutilized fresh maize in the preparation of porridge to contribute toward complementary feeding of children, and reductions in pre‐harvest losses. Fresh maize was harvested at different stages of maturity, blanched, smoked, and sun dried before milling into flours that were blended with soy flours for preparation of test porridges. The test flours were analyzed using the Association of the Official Analytical Chemists (AOAC) methods to determine their nutrient composition before preparation of the porridges. A trained consumer panel of 12 people, mothers and nursery school children tasted the porridges to rank acceptability and preference. Analysis of nutritional data showed that the test flours contained similar amounts of proteins, fats, and carbohydrates as the commonly used dried maize–soy flour blends. All the test porridges were generally accepted by the mothers and children due to the unique smoky and roasted aroma, brown color and the sweeter flavor even without the addition of sugar. Grainy texture and the presence of residues were the only unacceptable attributes in some of the test porridges. In conclusion, fresh maize–soy floor blends can be potentially used in complementary feeding of children at home and school as an alternative to other traditional maize flours. Optimization and fortification can help make the flours nutrient‐dense and most appropriate for child feeding at scale.

## 1 INTRODUCTION

Maize is consumed widely in the world. In Malawi and other countries in sub‐Saharan Africa, maize is the staple food eaten in different forms as porridge during breakfast or stiff paste for lunch and supper. Different groupings and tribes use different names for the stiff paste in the region (e.g., *nsima*,* sadza, pap, ugali, and poshto*). Fresh and immature maize is one of the maize products that is highly traded and utilized in almost all households in Malawi. While maize remains an important staple, utilization of fresh maize is limited to boiling and roasting. A lot of fresh maize is harvested for consumption, but due to its perishability and lack of processing techniques, leftovers get wasted.

To extend the shelf life, women preserve fresh maize through blanching and drying by open sun and using smoke from firewood or charcoal. Fresh maize preserved this way is traditionally known as *Viselera* in some parts of Malawi. Williamson (1975). It is shelf stable, widely accepted and mainly eaten as a boiled snack by all family members in times of scarcity. Despite its popularity particularly in rural Malawi, there is no known industrial application of the product and, as a result, it remains un‐investigated and not well understood. Since fresh maize has a wide acceptance, there is need to consider its use in a wider range; for example, by processing it into flour and developing new products in a bid to reduce pre‐harvest losses and diversify diets. Currently, diets intended for feeding of children less than five are less diversified and predominated by native dried maize. Although highly consumed, maize has low protein (9.42%) and is deficient in essential amino acids, such as lysine and tryptophan, but has fair amounts of sulfur‐containing amino acids (methionine and cysteine) according to FAO (1992). To improve the protein quality, maize is consumed together with other protein sources such as legumes, milk, soybeans, and amaranth seeds and leaves, which are relatively rich sources of lysine and tryptophan, but are low in sulfur amino acids contained in maize (Food and Agriculture Organization of the United Nations, 1992). In Malawi and other countries in sub‐Saharan Africa, maize–soy blends are produced commercially and locally promoted by governments and their development partners for feeding young children and adults in schools, hospitals, orphanage centers, and refugee camps (Ng'ong'ola‐Manani, Mwangwela, Sch€uller, Østlie, & Wicklund, 2014; Lawless & Heymann, 2010).

The aim of this study was to assess the nutritional content of smoked and sun‐dried fresh maize flours and evaluate consumer acceptability of porridges prepared to contribute toward the development of foods for complementary feeding of young children in the country. Being the first of its kind, the present study will contribute valuable data to the food composition database currently under development in the country in addition to informing the public about other possible uses of fresh maize besides boiling and roasting. Given that child under nutrition is high in Malawi with 37 in every 100 children under 5 years suffering from stunting and 11.7 percent underweight (National Statistical office 2015‐2016) the study will contribute valuable information for decision making by policy makers, nutritionists, and community workers on how best maize can be used differently for child feeding.

## 2 MATERIALS AND METHODS

### 2.1 Preparation of fresh maize preserves and maize preserve flours

**Figure 1:**
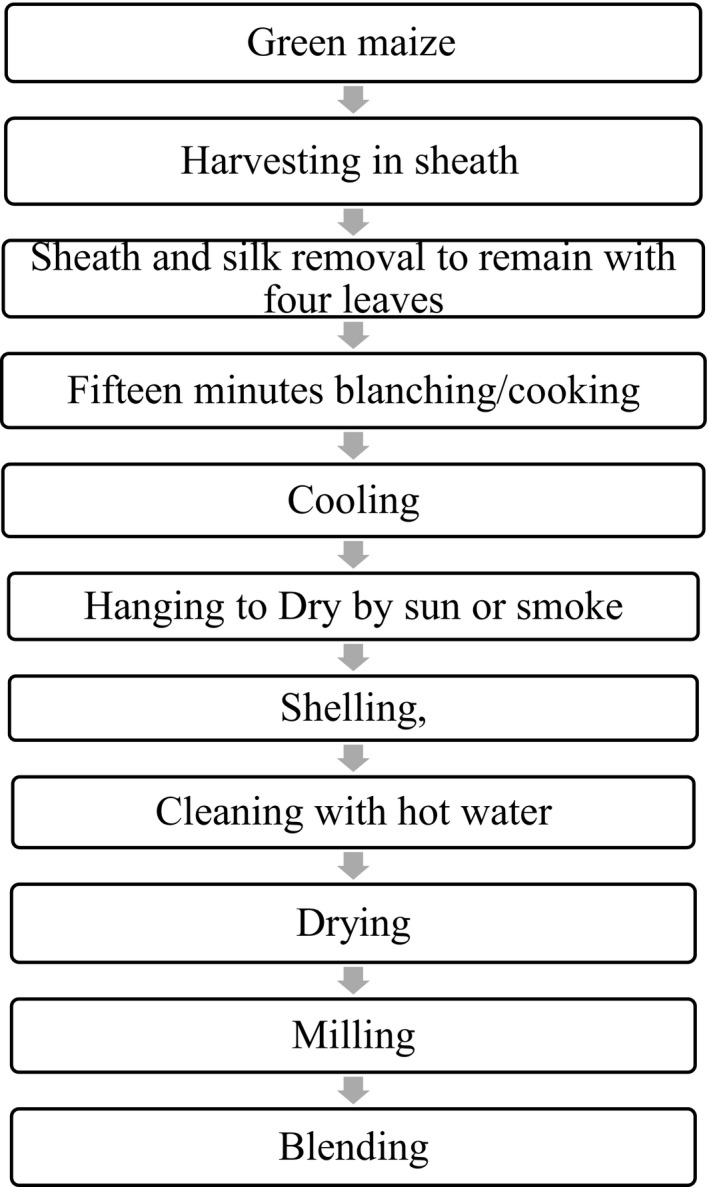
Process flow chart for the preparation of fresh maize preserves and flour

**Figure 2:**
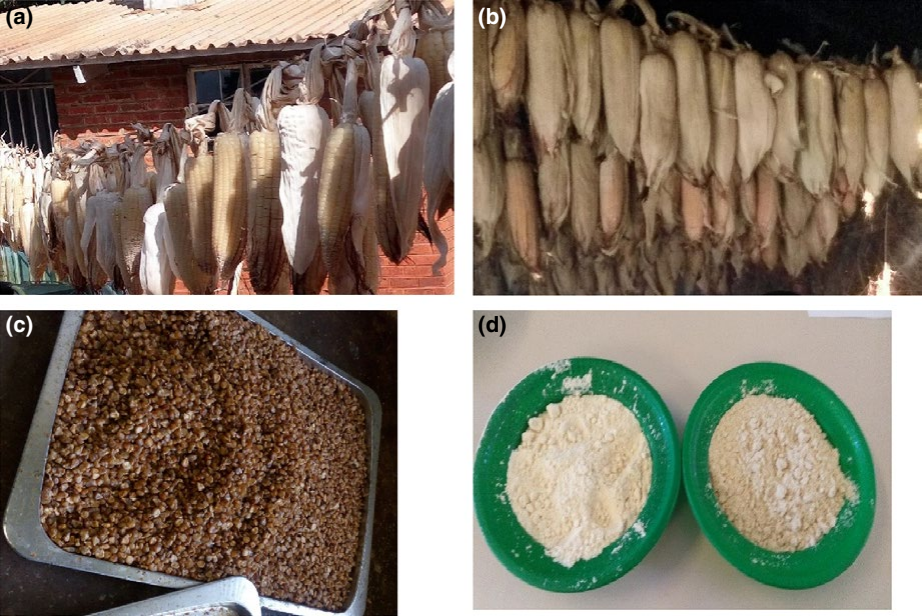
Picture showing blanched fresh maize during drying and fresh maize–soy blends. (a) open sun drying of blanched fresh maize; (b) fresh maize being smoke dried; (c) shelled smoked fresh maize preserve; (d) fresh maize–soy blends

Fresh maize preserves were prepared with slight modifications of method proposed by Dauda (2014). Fresh green maize harvested at different stages of maturity (20DAP, 27 DAP, 40 DAP) was blanched in boiling water for 15 min and cooled in cold water immediately. The sheaths were then removed before tying the cobs to poles in a wooden smoke kitchen and open sun dried (Figure**** 1). After drying, the blanched and dried maize was milled into flours that were blended with soy and used to prepare the test porridges. Figure**** 2 depicts the unit operations followed in the processing of the flours.

### 2.2 Preparation of the control porridge

Maize dried in the field after full maturity was milled and combine with soy to prepare control porridges for the study as traditionally done by Malawian women.

### 2.3 Preparation of flour blends for test porridges

A total of ten flour blends were formulated by blending 80 g of either dried fresh maize flour or native maize flour with 20 g boiled or roasted, dried, and milled soy. Flours formulated were as follows: traditional maize flour + roasted soy flour(MRS); maize + Boiled soy (MBS); smoked milk stage maize + roasted soy(SMRS); smoked physiological maturity fresh maize flour roasted + soy(SMPRS); smoked milk + boiled soy (SMBS); smoked dent + boiled soy (SMDBS); smoked physiological maturity + boiled soy (SMPMS); smoked dent + roasted soy (SMDRS); sun‐dried physiological maturity + boiled soy (SNPMBS); and sun‐dried physiological maturity roasted soy (SNPMR). The test porridges were prepared by adding the flour to warm water until a soft paste was formed using high heat for 10 min and simmering at low heat for 50 min to avoid burning. Homemade maize–soy flour blends (locally known as *Likuni phala*) comprising of flours from dried fully matured maize and roasted soy were prepared and cooked in a similar manner as the control.

### 2.4 Proximate analysis

Six flours (smoked milk stage flour (SMF), smoked dent stage flour (SMDF), smoked physiological maturity (SMPMF), sun‐dried milk stage flour (SNMF), sun‐dried dent flour (SNDF), sun‐dried physiological maturity flour (SNPMF), and the dried maize flour (NDMF) made from fresh maize harvested at different stages of maturity (20DAP, 27DAP, and 40 DAP), blanched and dried by open sun drying and smoke drying were subjected to proximate analysis suggested by AOAC (2002) to determine their nutrient composition.

### 2.5 Descriptive sensory analysis

The researcher recruited a panel of 10 trained judges consisting of five women and five men chosen purposively from the University staff and students. The judges undertook a rigorous descriptive analysis of six representative fresh and normally dried maize–soy blends (MRS, MBS, SNPMRS, SNPMBS, SMPMRS, and SMPMBS) before other sensory evaluation tests were conducted. Only judges with the ability to differentiate four basic tests (salty, sour, sweet, and bitter) in the triangle test used were recruited among the 20 tasters who expressed interest. Training of the sensory panel was done as described by Lawless and Heymann (2010) and Ng'ong'ola‐Manani et al. (2014). The exercise helped to generate consensus regarding terms to be used to describe porridge samples of this study.

#### 2.5.1 Sample preparation and presentation

The six prepared warm porridges were presented to the panelists in the identical coded containers at random to avoid bias and obtain valid responses for analysis and interpretation.

#### 2.5.2 Descriptive sensory analysis

Descriptive sensory evaluation was conducted in triplicate using randomized blocked design in a well‐ventilated, aerated, and lighted class room for 3 days after the seven‐day training. The panelists evaluated the samples, rinsing their mouth with clean tap water were necessary in the process. Scoring was done using a nine‐point hedonic scale ranging from 1 “dislike extremely” to 9 “like extremely.” Attributes with statistically significant scores were taken for further analysis as described by Tomic et al. (2009).

### 2.6 Consumer acceptability study

#### 2.6.1 Acceptability study by babies and nursery school‐going children

The consumer acceptability study engaged babies and nursery school children, being the ones who traditionally consume porridge as their daily food. Based on the recommendations of the trained descriptive panel, the babies and nursery school‐going children tasted all the seven test porridges over the seven‐day period spread within 2 weeks. Given that children have small stomachs; one porridge was tasted at a time to get reliable consumer acceptability results. Mothers were given 150 ml of porridge to feed their children until they were full in this regard. During the feeding, facial expressions were observed using a five‐point Likert type facial smiley scale (Lawless & Heymann, 2010), with a score of one meaning that the child disliked the porridge, while a score of five meant that she liked it extremely. The mothers were instructed to present any remaining porridge to the research assistants who weighed and indicated the amount of plate waste in grams as part of the acceptability results.

#### 2.6.2 Consumer acceptability tests by the mothers

Mothers are the ones who cook porridge at home for children. After feeding young children as part of sensory evaluation for this study, mothers were asked to choose the most acceptable porridge based on color, taste, and texture using a nine Likert type hedonic scale. The seven test porridges evaluated by the children were the ones given to the mothers of this study. On each tasting day, the mothers were asked to taste the porridge they were feeding their children and rate it using the aforesaid scale that ranged from 1 “dislike extremely” to 9 “like extremely.”

#### 2.6.3 Preference ranking tests by the mothers

Besides the consumer acceptability test, the mothers were also asked to taste and rank their preference of the test porridges using a seven‐point Food Action Rating Scale (FACT) sheet (Table 1) as described by (Lawless & Heymann, 2010).

**Table 1:** Food Action Scale Rating Scale used in the preference ranking test by the mothers

Description	Code
I would feed this porridge to my child every opportunity I had	7
I would frequently feed this porridge to my child	6
I like this porridge and would feed it to my child now and again	5
I would feed this porridge to my child if available but would not go out of my way	4
I don't like this porridge but would feed it to my child on occasion	3
I would feed this porridge to my child only if there were no other choices	2
I would feed porridge to my child only if I were forced to	1

### 2.7 Statistical analysis

Sensory and nutrient analysis data were entered and analyzed in SPSS version 20 (Statistical Package for Social Sciences, Inc., Chicago, IL, USA). Descriptive statistics, such as means, frequencies, and percentages, were generated and used to describe the findings. Tests for robustness of means were done using Games‐Howell tests. Principal component analysis (PCA), PSLR was used to determine variations and bring out strong patterns in the dataset as described by (Ng'ong'ola‐Manani et al., 2014).

## 3 RESULTS

### 3.1 Socio‐demographic characteristics of the study subjects

The study subjects were 82 mothers and 113 nursery school children 3–5 years of age. Porridge is the most common food for young children while mothers are the main decision makers on what kind of porridge to prepare for their children. Table**** 2 presents more details about the socio‐demographic characteristics of the study subjects.

**Table 2:** Socio‐demographic profile of the study subjects(*n* = 89)

Characteristic	Frequency	
	*n*	%
Mothers age		
Less than 20	16	19.5
20–24 years	42	51.2
25–29 years	18	22.0
30–34 years	2	2.4
40–45 years	4	4.9
Marital status of the mothers		
Single	6	7.3
Married	74	90.2
Divorced	2	2.4
Level of education of caregiver		
None	4	4.9
Primary	60	73.2
Secondary	14	17.1
More than secondary	4	4.9
Occupation of caregiver		
Farming	56	68.3
Casual labor	2	2.4
Paid job	6	7.3
Business	14	17.1
Others	4	4.9
Number of children in a family		
1	38	46.3
2	26	31.7
3	10	12.2
4	4	4.9
5	2	2.4
6+	2	2.4
Knowledge about maize–soy blends		
Yes	56	68.3
No	26	31.7
Does the mother feed child maize–soy blends		
Yes	42	51.2
No	40	48.8
Source of maize–soy blend		
Buy	20	24.4
Process	22	26.8
No maize–soy blend feeding	42	51.2
Consumption of fresh maize preserves		
Yes	20	24.4
No	62	75.6
Roasted maize	34	42.5

### 3.2 Nutrient content of test flours

Table**** 3 shows the results for nutritional composition of fresh and traditional dried maize flours. In general, the flours prepared from dried and smoked fresh maize have shown similar amounts of proteins, fats, zinc, and iron with traditional dried maize, but higher amounts of ash and iron compared to maize flour from traditionally dried maize. The amounts of carbohydrates were almost similar to those of normally dried maize, but with lower levels of potassium.

**Table 3:** Proximate nutrient composition of fresh maize flours dried by smoke and sun drying, and the control (normally dried maize flour)

Nutritional content	Sun dried			Smoke dried			Field dried	*p*‐value
	SNMF	SNDF	SNPMF	SMMF	SMDF	SMPMF	NDMF(Control)	
Moisture	3.6 ± 2.9	6.2 ± 4.2	7.39 ± 0.e	6.0 ± 0.1	5.6 ± 0.3	5.0 ± 0.4	7.9 ± 0.3	0.331
Ash	7.1 ± 0.8^d^	8.5 ± 0.9^e^	6.8 ± 0.0.3^c^	6.7 ± 1.2^c^	6.2 ± 0.6^c^	3.2 ± 3.2^b^	1.6 ± 0.4^a^	0.034^a^
Protein	9.5 ± 1.4	7.3 ± 5.0	13.7 ± 0.2	11.5 ± 1.3	10.7 ± 0.2	10.3 ± 1.0	11.7 ± 2.2	0.230
Fat	6.5 ± 0.3	7.8 ± 0.4	7.2 ± 2.2	5.3 ± 0.8	6.4 ± 0.6	7.0 ± 2.1	7.2 ± 1.1	0.423
Carbohydrates	73.3 ± 6.3	70.2 ± 1.4	64.9 ± 0.7	70.6 ± 2.2	71.1 ± 6.3	74.4 ± 6.3	71.6 ± 4.2	0.216
Zinc mg/g			3.7 ± 0.7	3.3 ± 0.2	3.5 ± 0.4	3.5 ± 0.6	3.6 ± 0.0	0.691
Iron mg/100 g			5.8 ± 1.2^b^	3.2 ± 0.0^a^	5.8 ± 0.0^b^	7.3 ± 0.0^c^	5.6 ± 0.0^b^	0.002^a^
Potassium g/100 g			360.3 ± 31.5^c^	392.4 ± 21.8^b^	337.0 ± 24.0^d^	312.5 ± 10.6^e^	616.4 ± 0.0^a^	0.000^a^
Phytate g/100 g			0.56 ± 0.48^a^	0.53 ± 0.5^a^	0.57 ± 0.49^a^	0.42 ± 0.36^a^	0.24 ± 0.36^a^	0.972

a Results are mean values of three determinations. Mean values with same letters are not significantly different.

### 3.3 Descriptive sensory analysis

**Figure 3:**
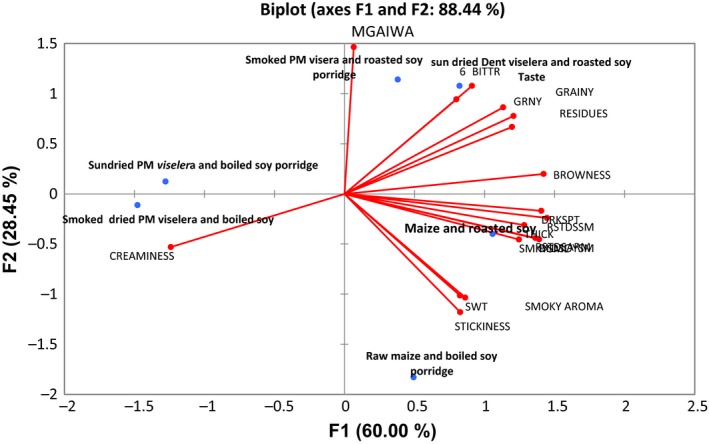
Bi‐plot showing the relationship between porridge samples and attributes in space of maize–soy blends as evaluated by descriptive trained panel. *Note*: BITTR = bitterness, AFST = aftertaste, GRNY = grainy, RES = residues, BRWN = brownness, DRKSPT = dark spots, THICK = thickness, SMYARM = smoky aroma, STICKY = stickiness

Six test porridges were evaluated by the trained panel of 12 people before assessments by the mothers and children. Attributes generated by the sensory panel to describe different test porridges and their scores are given in Table**** 4. Figure**** 3 presents a bi‐plot to show sensory loadings and variations for the porridges generated from principal components analysis (PCA).

**Table 4:** Attributes generated to describe different maize–soy porridges

Attribute	Raw maize plus roasted soy	Raw maize plus boiled soy	Smoked *viselera* plus roasted soy	Sun‐dried *viselera* plus boiled soy	Smoked *viselera* plus boiled soy	Sun‐dried *viselera* plus roasted soy	*F*	Pr > F
Creaminess	4.8 ± 3.0^c,b^	4.7 ± 3.1^c,b^	4.0 ± 2.7^c,b^	5.4 ± 2.8^b,c^	6.3 ± 2.7^a,b^	3.8 ± 2.6^c,b^	3.640	0.004
Brownness	6.2 ± 2.6^a^	5.6 ± 2.7^b^	6.5 ± 2.2^a^	2.8 ± 2.3^c^	2.8 ± 2.0^c^	6.0 ± 2.3^a^	15.814	<0.0001
Graininess	5.8 ± 3.1^b^	4.1 ± 2.7^b^	5.3 ± 2.3^b^	4.0 ± 2.0^b^	3.8 ± 2.4^c,a^	6.1 ± 2.^a^	4.564	0.001
Dark spots	7.2 ± 2.1^a^	5.3 ± 2.5^b,a^	4.6 ± 2.5^c,b,d,a^	3.2 ± 2.3^d^	2.4 ± 1.7^d^	5.6 ± 2.6^a,b,c^	17.034	<0.0001
Smoky aroma	5.6 ± 2.5^a^	6.5 ± 2.7^a^	4.8 ± 2.8^a,b^	3.3 ± 2.7^b^	4.8 ± 2.6^a,b^	4.4 ± 2.7^b^	5.184	0.000
Roasted soy Aroma	6.5 ± 2.4^a^	5.8 ± 3.0^a^	4.4 ± 2.5^a,b,c^	3.3 ± 2.8^b,a,c^	3.6 ± 2.2^c,a,b^	5.6 ± 2.7^a^	7.126	<0.0001
Soy aroma	5.7 ± 2.8^a^	5.7 ± 2.9^a,c^	4.4 ± 2.5^a,b,c^	2.9 ± 2.1^b,c^	3.3 ± 2.2^c^	5.2 ± 2.5^a^	7.330	<0.0001
Mgaiwa smell	5.0 ± 2.4^a^	3.9 ± 2.5^a^	6.0 ± 2.3^a^	5.4 ± 3.1^a^	4.9 ± 2.7^a^	5.8 ± 2.4^a^	2.494	0.033
Sweet taste	5.8 ± 2.8^a^	6.5 ± 2.6^b,a^	3.7 ± 2.0^b,a,c^	4.6 ± 2.6^a,b,c^	3.4 ± 2.6^c,b,a,^	4.9 ± 2.7^a^	6.677	<0.0001
Bitter taste	4.3 ± 2.9^a^	3.7 ± 2.8^a^	4.9 ± 2.9^a^	4.0 ± 2.9^a^	3.5 ± 2.5^a^	4.7 ± 2.7^a^	1.038	**0.397**
Residues	5.8 ± 2.8^a^	3.9 ± 2.5^a,b^	5.0 ± 2.4^a,b^	3.7 ± 2.4^b,a^	3.8 ± 2.5^a,b^	5.6 ± 2.4^a^	3.691	0.003
Aftertaste	4.3 ± 2.7^a^	4.4 ± 2.9^a^	5.2 ± 2.8^a^	4.0 ± 2.9^a^	4.3 ± 3.1^a^	5.5 ± 2.5^a^	1.268	**0.280**
Stickiness	5.2 ± 3.1^a^	6.3 ± 2.8^a^	4.3 ± 2.4^a^	4.1 ± 2.5^a^	3.8 ± 2.6^a^	4.2 ± 2.6^a^	3.735	0.003
Thickness	6.6 ± 2.6^a,^	6.0 ± 2.7^a^	5.4 ± 2.8^a,b^	5.0 ± 2.6^a,b^	3.5 ± 2.4^b^	5.6 ± 2.7^a^	4.768	0.000

1 = not sweet, 9 = very sweet (for every attribute) Means not sharing a superscript within a row are significantly different from each other.

### 3.4 Acceptability of the test porridges by the mothers

Table**** 5 presents results from acceptability tests of the seven porridges. There were significant differences (*p* < 0.05) in the general acceptability of the prepared maize–soy blends.

**Table 5:** Acceptability results for control and test porridges by the mothers^a^

Type of Porridge	Color	Smell	Taste	Overall Acceptability
Normal field dried maize + roasted soy	6.86 ± 2.14^a^	6.78 ± 2.23^a^	6.78 ± 2.18^a^	4.88 ± 1.90^a^
Normal field dried maize + boiled soy	6.70 ± 2.05^a^	6.92 ± 1.83^a^	7.48 ± 1.73^a^	5.08 ± 1.58^a^
SmSmoked milk stage maize + roasted soy	5.04 ± 2.32^b^	4.70 ± 2.46^b^	4.84 ± 2.29^b,a^	3.22 ± 2.09^a,b^
Smoked physiological + roasted soy	7.00 ± 1.78^a,b^	7.16 ± 1.39^a,b^	7.04 ± 1.12^a^	5.28 ± 1.26^b^
Smoked milk stage + boiled soy	7.15 ± 1.84^a^	7.50 ± 1.67^a,b^	7.50 ± 1.73^a^	5.90 ± 1.29^a^
Smoked dent + boiled soy	4.04 ± 1.87^b^	3.62 ± 1.96^b^	3.76 ± 1.91^b^	2.48 ± 1.46^b,c,a^
Smoked physiological maturity + boiled soy	5.86 ± 2.11^a^	5.64 ± 2.26^a,b^	5.66 ± 2.05^a^	3.88 ± 1.79^a^

a *Note*: 1 = dislike extremely and 9 = like extremely (for taste, smell, and flavor). For Overall acceptability scale of 1 to 7 used. 1 = I will only feed my child if forced to and 7 (I will feed my child every other opportunity I get).

### 3.5 Observed consumption and left overs by children

Similarly, the study found no significant differences in the acceptability and preference of the different maize–soy blends presented. Observations of plate waste showed that all the test porridges were well‐accepted by the children, with exception of dent fresh maize–soy blend (SMDBS) porridge that had some leftovers. No significant differences (*p* < 0.05) were also recorded on the amounts of leftovers.

### 3.6 Sensory results from the mothers and children

**Figure 4:**
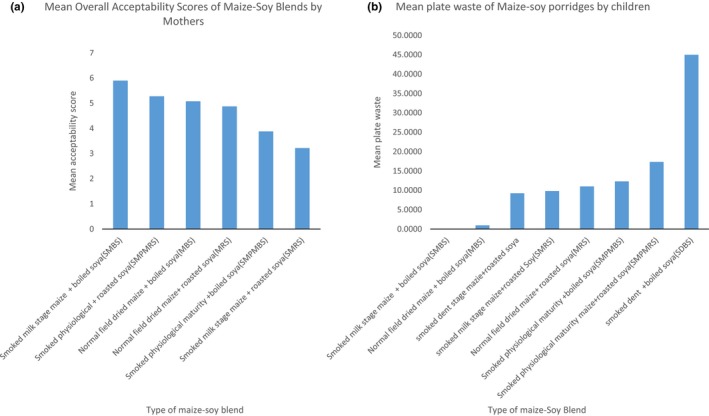
Pareto charts showing preference by mothers (a) and percentage consumption by children (b) for the seven porridges

To understand the differences in acceptability of the developed porridges between mothers and children, Pareto charts were developed presenting samples from higher liking to the lowest liking (Figure**** 4a and b).

### 3.7 Drivers of acceptability and preference

**Figure 5:**
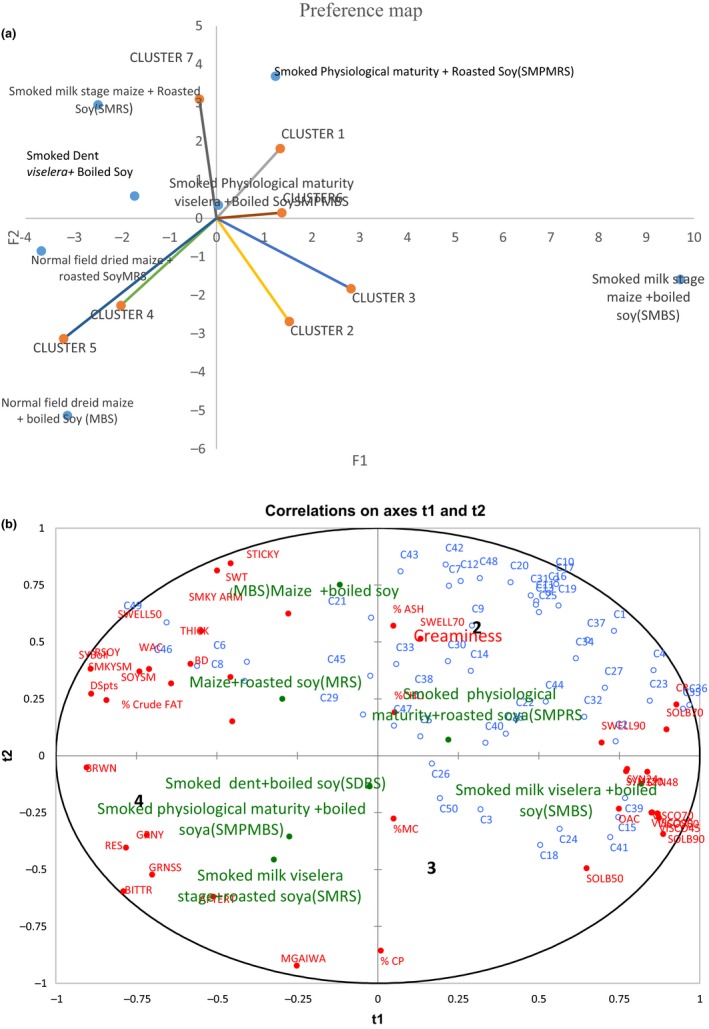
(a) Combined preference map showing consumer clusters and products they liked.
Sensory, chemical, and functional properties of different porridgesConsumers distribution in spaceFresh corn preserves (FCSBS) and Normally dried corn–soy blends(CSB)(b) Correlations map showing the attributes which influenced consumer preferences of different porridge blends

To understand drivers of liking of different porridge blends, partial least square regression analysis (PLSR) and external preference mapping were performed on the data from consumer acceptability study. Figure**** 5a and b summarizes the results obtained.

## 4 DISCUSSION

Porridge is the most common food for young children. Nevertheless, its energy and nutrient content is often too low to meet their needs fully due to the high starch content of staple foods, such as maize, millet, sorghum, and cassava. Food analysis data from this study (Table 3) showed that fresh maize flours had similar nutrient composition as common flours from dried maize reported by Food and Agriculture Organization of the United Nations (1992), in the range of 64.9–74.4% for carbohydrates, 9.51%–13.7% protein and 5.3%–7.8% fat per 100 g of the flours. These results were higher than those reported by Chukuma, Taiwo, and Udouso (2016) for roasted and boiled fresh maize. Except for moisture content, which was lower than the recommended standard by the World Food Programme (WFP, 2012) , various nutrients were above the World Health Organisation's (WHO) minimum thresholds. These results mean that the test flours (fresh maize preserves) can be used equally in the cookery of porridge for children. Ways to make the porridges more energy and nutrient‐rich should include adding energy‐rich, such as oil and butter/ghee, and nutrient‐rich foods (such as flours of groundnut and other legumes, sunflower seed and fish or vegetable mash) to the porridge. In addition, Malawi does not have own Food Composition Tables and continues to rely on data from other countries, making the nutrient results from this study all the more important.

We found mixed results for sensory evaluation by mothers and children. All children liked the test porridges with exception of SMDB that was also least liked by the mothers. Nevertheless, the MBS porridge ranked third most acceptable by the mothers was second most preferred by the children, with only 1% plate waste (Figure 5). This result means that although the decision on which porridge and complementary foods to serve to babies is made by mothers, the likes may differ. Descriptive tests by trained panelist already showed that MBS, SMPMRS, and SMBS had high scores in taste, flavor and color while SMPBS, SDBS, and SMRS had lowest scores in the same attributes. Although MBS had high flavor, taste, and color based on the evaluation by the mothers, it paradoxically had much lower acceptability scores along with SMPMBS **(**Table**** 5 **)**. Sensory attributes contributing to the acceptability and preference of the other maize porridges were creaminess and sweetness (Figure 3). Bi‐Plots of the attributes and samples (Figure 3) loaded most of the test porridges to have similar sensory attributes which were located in the same space, for example, PCA 1 and 2 explained 88.54% of the variation. Creaminess was negatively correlated with brownness on the bi‐plot. Smoky flavor, sweetness, and roasted soy aroma were drivers of liking of both the native and fresh maize porridges while graininess, poor aromas, and residues were the major reasons for the tasters to dislike other porridges mentioned above. Processing of maize while fresh and the use of smoking contributed to enhanced flavors that were not found in the control porridge.

In this study, nearly all the mothers were aware of the traditional practice of preserving fresh maize in the villages for reconstitution in future, but none of them had ever done so before. The majority of them claimed preparing children's porridge using dried maize–soy flour blends (locally known as *Likuni Phala*). As already explained, when asked to evaluate the test porridges whose maize was harvested while fresh and smoked, the mothers of this study found the porridges acceptable and preferred most the unique smoky and sweet flavors even without the addition of sugar. Cluster analysis and external preference mapping (Figure 5a and b) provided a clear picture for the drivers of acceptability and preference. According to these figures, clusters 5 and 4 liked MRS, MBS porridges characterized by sweetness, creaminess, and boiled soy aroma. Cluster 7 liked SMBS, the porridge liked most by both children and their mothers. The SDBS porridge, which was liked least by all groups of evaluators including the descriptive panel, was not located near any attributes on the preference map, suggesting that the reasons for dislike were outside the descriptions provided. The study observed no gender differences in the acceptability of the test porridges among children. Age had an effect as well on the amount of porridge left on the plate with younger children eating less than their older counterparts as presumed.

One main contribution of the present study is its contribution to the limited literature on the processing and value addition of fresh maize. The study has demonstrated that flours prepared from fresh maize can blended successfully with soy flours and used to cook porridge for children. The study has also provided research‐based evidence that minimizing wastage can help improve availability and access of food in addition to increasing diversity of diets in country. We observed an increase in school attendance after the study commenced. This result means such porridges can be used at scale in community school feeding programmes and positive deviance hearth sessions meant for undernourished children.

Rigorous analysis of the contemporary literature shows that previous studies have only investigated sensory acceptability of fresh maize or suitability of other maize varieties for fresh maize consumption (Harriman, Ngwuta, Onyishi, & Nzee, 2013); Alamu, Olaofe, Maziya‐Dixon, & Menkir, 2014); Chukuma et al., 2016) and the acceptability of reconstituted fresh maize preserves (Dauda, 2014; Williamson, 1975). Different researchers have also investigated the utilization of flours from dried maize–soy blends (CSBs) in local diets for children and found them well acceptable (Achidi et al., 2016 *;* Alamu, Maziya‐Dixon, Popola, Gondwe, & Chikoye, 2016; Amegovu et al., 2014; Cheryan, Mc Cune, Nelson, & Ferrier, 1979; Chukuma et al., 2016; Emire & Buta, 2015; Fikiru, Bultosa, Forsido, & Temesgen, 2016; I‐Brockdorf et al., 2015; Kalimbira, Mtimuni, & Mtimuni, 2004; Kehlet, Kæstel, Hausner, Bredie, & Allesen‐Holm, 2011; Muhimbula, Issa‐Zacharia, & Kinabo, 2011; Ng'ong'ola‐Manani et al., 2014). This study is the first of its kind to formulate and test the acceptability and preference of porridges prepared from smoked and dried fresh maize–soy blends. Given that every country is different with respect to diet and lifestyles by virtue of cultural, socio‐economic and contextual factors, there is still a strong need for more research in a wider variety of countries in sub‐Saharan Africa to generate country‐specific data for guiding nutrition programming and cookery of complementary foods.

The study was not without limitations. The green maize season falls in rainy months which generally presented problems in drying, with some of the maize cobs developing moulds. Considering the need to get valid responses and since children have small stomachs, the study designed to carry out the sensory evaluation of the seven test porridges within two weeks, relying on the same cohort of subjects to allow easy comparisons of the results. In real life situation, this period was longer than anticipated; some mothers dropped out in the process. Besides this, the cessation of the study led to frustrations of many children who got used to eating the porridges in the evaluation exercise. For example, when the study began the children were very excited taking it as one of the school feeding programmes and the numbers kept on increasing, for example, from 27 kids at the beginning to 111 at the end, only to realize that it ended sooner than they expected. No analysis of poly‐aromatic hydrocarbons(PAH) in the smoked maize products was conducted in this study. However, literature reviewed from other studies **(**Table**** 6 **)** reveals the wide nature of PAH in foods and the lack of a specific legislation on allowable PAH levels in maize. Although an allowable limit has been set by the European Union for baby products, none of such legislation exists for PAH in foods in Malawi.

**Table 6:** Poly‐aromatic hydrocarbons found in some foods

Product name	Country	Types of PAH FOUND	References
Corn submitted to drying by firewood	Brazil	Seven compounds; fluorene, phenanthrene, anthracene, fluoranthene, pyrene, benzo(a)anthracene, and chrysene were detected.	De Lima et al. (2016)
Toasted Bread(18 samples)	Kuwait	No B[a]P was detected in ten of eighteen samples as well as in original white and brown wheat flour. In eight samples, B[a]P varied from 2.83 to 16.54 g/kg. B[a]A, CHR, B[b]FA, B[k] FA, IP, DB[a,h]A, and B[ghi]P concentrations were found to be less than 10.0 g/kg. Total PAHs were varied in the range 1.06–44.24 g/kg and 3.08–278.66 g/kg for H‐PAH and L‐PAH, respectively.	Al‐Rashdan, Helaleh, Nisar, Ibtisam, and Al‐Ballam (2010)
Rice	Not indicated	Benzo(a) pyrene, the marker used for evaluating the carcinogenic risk of PAHs in food, was not detected in rice samples. However, Naphthalene, phenanthrene, and fluoranthene were detected in the rice samples analyzed.	Escarrone et al. (2014)
Liquid smoke flavor (11 samples) and some smoked foods (44 samples of smoked foods like bacon, loin, turkey, sausage, ox rib)		Benzo[a]pyrene was found in 73% of the liquid smoke flavor samples analyzed. From the total of 44 smoked food samples analyzed, benzo(a)pyrene was detected in 23 samples (52%).Anthracene and fluoranthene, non‐carcinogenic polycyclic aromatic hydrocarbons, were found in almost all the samples analyzed. Benzo[ghi] perylene, 3,4‐benzofluoranthene and 1,2,3,4‐dibenzopyrene were not found in any of the 55 samples analyzed.	
Smoked foods including turkey, pork, chicken, beef, and fish products and		Total PAH concentrations Smoked meat products;(a) 2.6 μg/kg in a cooked ham, 29.8 μg/kg in grilled pork chops. In fish products ranged from 9.3 μg/kg in smoked shrimp to 86.6 μg/kg in smoked salmon.(b) Total concentrations of the carcinogenic PAHs (benzo [a]anthracene, benzo [b]fluoranthene, benzo [a]pyrene, di‐benzo [a, h] anthracene, and indeno [1,2,3‐c, d] pyrene) ranged from non‐detectable in several meat products to 7.4 μg/kg in grilled pork chops, and from 0.2 μg/kg in trout to 16.0 μg/kg in salmon.	Gomaa, Gray, Rabie, Lopez‐Bote, and Booren (1993)
Eighteen commercial liquid smoke flavorings and seasonings		Total PAH concentrations (6.3 to 43.7 μg/kg); carcinogenic PAHs (0.3 to 10.2 μg/kg)	Gomaa et al. (1993)
Smoked meat and meat products(Thirty‐eight samples) smoked fish (39 samples)	Sweden	Nine samples of smoked meat (produced by traditional “sauna” smoking, where the food is directly exposed to hot smoke from a burning log fire) showed high BaP levels ranging from 6.6 to 36.9 μg/kg, exceeding the 5.0 μg/kg maximum level for smoked meat and fish established by the European Commission (Regulation [EC] No 208/2005).Six samples of smoked fish had BaP levels exceeding 5.0 μg/kg, the concentrations ranging from 8.4 to 14.4 μg/kg. Samples of meat and fish smoked by indirect technique, using smoke from an external smoke generator, all had BaP levels below the limit of quantification, that is, 0.3 μg/kg	Wretling, Eriksson, Eskhult, and Larsson (2010)
Toasted bread (direct toasting (flame‐toasting, coal‐grilling or gas oven‐toasting) or indirect toasting (electric oven‐toasting)		None of electric oven and toaster bread were polluted; samples toasted by charcoal and flame grilling contained up to 350 μg/kg of total PAHs. Very low levels PAH levels were reported in several commercial toasted samples of bread. Benzo[a] pyrene ranged from no detectable to 0.23 μg/kg.	Salgueiro, Falcón, Carballo, and Gándara (2008).

## 5 CONCLUSION

Although fresh maize is consumed widely in developing countries of sub‐Saharan Africa, it is available only for a short period of time before drying in the garden. Preservation of fresh maize by smoking, oven and sun drying can reduce pre‐ and post‐harvest losses and ensure availability in times of scarcity to be used in various ways in the diets. Fresh maize–soy porridges, which have shown to be of high acceptability by young children of this study, can be prepared locally at scale to feed children at community level or nursery schools as part of positive deviance (PD) hearth sessions considering that pre‐school children are currently excluded from donor‐driven school feeding programmes in the country. While children's diets are decided by their mothers and other caretakers, the present study has found that sensory considerations of children can be different altogether from those of their mothers, confirming the importance of such studies even in real life intervention programs. Future studies should strive to develop different recipes for *Viselera* flours besides using it in the preparation of porridge to increase utilization in diets and benefits for communities. With the current focus on PAH in foods, there is need for future studies in this area and for Malawi Bureau of Standards to adopt legislation to establish maximum PAH levels in foods including baby products.

## CONFLICT OF INTEREST

The authors declare that they do not have any conflict of interests.

## ETHICAL STATEMENTS

This study was approved by the Ethics Committee at Lilongwe University of Agriculture and Natural Resources (LUANAR). Actual participation in the study was based on full consent from the mothers and nursery school administrators. The study participants were informed by the researcher about the voluntary nature of the study, highlighting on their rights to refuse participation, the right to skip a particular sensory session they did not want to participate, and the right to discontinue the exercise at any time. Each respondent was also assured of the confidentiality of the sensory responses given. The researcher and her assistants abided by their professional ethical conduct, such as neutrality, respect for tester's dignity, sensory perceptions, and data verification, throughout the period of data collection. Completed forms were not shared with anyone outside the study team.
